# The therapeutic landscape of tauopathies: challenges and prospects

**DOI:** 10.1186/s13195-023-01321-7

**Published:** 2023-10-06

**Authors:** Jeffrey L. Cummings, M. Isabel Gonzalez, Martyn C. Pritchard, Patrick C. May, Leticia M. Toledo-Sherman, Glenn A. Harris

**Affiliations:** 1 Chambers-Grundy Center for Transformative Neuroscience, Department of Brain Health, School of Integrated Health Sciences, University of Nevada, Las Vegas (UNLV), Henderson, NV USA; 2 Drug Discovery and Development Consultants Ltd., Cambridge, UK; 3 ADvantage Neuroscience Consulting LLC, Fort Wayne, IN USA; 4 MycRx Pharma, Austin, TX USA; 5 Rainwater Charitable Foundation, 777 Main Street, Suite 2250, Fort Worth, TX 76102 USA

**Keywords:** Alzheimer’s disease, Drug development, Frontotemporal dementia, Progressive supranuclear palsy, Corticobasal degeneration, Chronic traumatic encephalopathy, Tau, Tauopathy, Therapeutic pipeline, Pick’s disease, Argyrophilic grain disease, Primary age-related tauopathy

## Abstract

Tauopathies are a group of neurodegenerative disorders characterized by the aggregation of the microtubule-associated protein tau. Aggregates of misfolded tau protein are believed to be implicated in neuronal death, which leads to a range of symptoms including cognitive decline, behavioral change, dementia, and motor deficits. Currently, there are no effective treatments for tauopathies. There are four clinical candidates in phase III trials and 16 in phase II trials. While no effective treatments are currently approved, there is increasing evidence to suggest that various therapeutic approaches may slow the progression of tauopathies or improve symptoms. This review outlines the landscape of therapeutic drugs (indexed through February 28, 2023) that target tau pathology and describes drug candidates in clinical development as well as those in the discovery and preclinical phases. The review also contains information on notable therapeutic programs that are inactive or that have been discontinued from development.

**Supplementary Information:**

The online version contains supplementary material available at 10.1186/s13195-023-01321-7.

## Introduction

Neurodegenerative disorders (NDs) are a complex set of chronic brain diseases most of which begin in late life and progress from preclinical through mildly symptomatic to severe disease and death. Pathologically, NDs share the common feature of aggregated proteins that begin as soluble monomers, aggregate to high molecular weight oligomers, and fibrillize into protein aggregates. The NDs are differentiated neuropathologically by the specific protein that aggregates in the cells in the brain. Alpha-synuclein (⍺-syn) comprises the protein aggregates in Parkinson’s disease (PD) and dementia with Lewy bodies; progressive supranuclear palsy (PSP) and cortical basal degeneration (CBD) are characterized by pathologic accumulations of tau proteins; the huntingtin protein is present in cells in Huntington’s disease (HD); Alzheimer’s disease (AD) features neurofibrillary tangles (NFTs) comprised of phosphorylated tau (p-tau) proteins and extracellular beta-amyloid protein (Aβ) plaques; the most common protein aggregating in amyotrophic lateral sclerosis (ALS) is TAR DNA-binding protein 43 (TDP-43); and patients with frontotemporal dementia (FTD) may have aggregates of TDP-43 or tau protein [16, 30, 53]. The protein aggregates of NDs disrupt cellular function, metabolism, and survival, leading to neuronal cell death [34]. Although the recognized NDs have a primary aggregating protein, mixed proteinopathies are common and synergistic interactions among Aβ, tau, and ⍺-syn accelerate clinical decline [78]. Each ND has a distinctive neurogeography with disproportionate involvement of specific brain regions characteristic of each disorder. PD affects primarily the substantia nigra; dementia with Lewy bodies affects the substantia nigra, limbic system, and neocortex; PSP has a predominance of subcortical tau deposits but may have cortical deposits in some cases, and CBD typically has asymmetric cortical tau deposits; HD affects the caudate nucleus; AD begins in the hippocampus and spreads to other cortical regions; ALS affects lower motor neurons of the spinal cord and upper neuron upper motor neurons of the motor cortex; and FTD effects prefrontal and orbitofrontal cortex as well as anterior and medial temporal cortical regions [8, 19]. The anatomical localization of the greatest neuropathological impact produces a corresponding clinical syndrome [23]. PD features tremor, rigidity, and bradykinesia,dementia with Lewy bodies manifests hallucinations, parkinsonism, and fluctuating cognition; HD is characterized by a progressive choreiform syndrome and dementia; AD typically presents with an amnestic disorder that progresses to affect language, visuospatial, and executive function; ALS exhibits progressive muscle weakness, muscle fasciculations, and respiratory compromise; and FTD produces primary progressive aphasia or a behavioral variant with disinhibition and impulsiveness [35, 61, 89]. The classical presentation of each of these disorders has a recognizable phenotype, but overlapping clinical presentations are common [67].

Tau is the primary misfolded protein aggregating in numerous late-life NDs. Tauopathies are divided into primary tauopathies in which tau is the predominant protein abnormality and secondary tauopathies in which tau protein aggregates coexist with other protein abnormalities. Primary tauopathies include PSP, CBD, FTD with parkinsonism linked to chromosome 17 (FTDP-17), behavioral variant FTD, primary progressive aphasia (PPA; predominantly nonfluent forms), Pick’s disease (PiD), chronic traumatic encephalopathy (CTE), argyrophilic grain disease (AGD), aging-related tau astrogliopathy (ARTAG), globular glia tauopathy (GGT), tangle only dementia (TOD), and primary age-related tauopathy (PART) (Table 1) [21, 40, 57, 70, 74, 83, 90]. Together these primary tauopathies affect millions of individuals with no currently available therapeutic alternatives that address the primary tau-related pathology and concomitant progressive neuronal degeneration [98]. A key molecular differentiating feature among the tauopathies is the presence of 3R or 4R splice variants. AD, CTE, TOD, PART, and PPA are 3R/4R tauopathies,GGT, ARTAG, AGD, CBD, and PSP are 4R tauopathies; PiD is a 3R tauopathy; behavioral variant FTD is primarily a 3R tauopathy; and FTDP-17 can express 3R, 4R, or mixed 3R/4R splice variants (Table 1) [83].

**Table 1:** Examples of primary and secondary tauopathies

**Tauopathy type**	**Name**	**Tau isoforms**	**Pathology**	**Localization**	**Presentation**
Primary	Progressive supranuclear palsy (PSP)	4R	NFTs, tau deposits in astrocytes	Midbrain, basal ganglia, diencephalon	Supranuclear vertical ophthalmoplegia, pseudobulbar palsy, and dementia
	Corticobasal degeneration (CBD)	4R	NFTs, coiled bodies, argyrophilic threads, astrocytic plaques	Primary motor cortex, basal ganglia, white matter	Progressive, asymmetric apraxia and akinetic-rigid syndrome
	Frontotemporal dementia with parkinsonism linked to chromosome 17 (FTDP-17)	4R, 3R or 3R/4R	Neuronal and glial tau deposits	Frontal and/or temporal lobe	Language-related dementia syndromes termed primary progressive aphasia with preserved memory
	Pick’s disease (PiD)	3R	Pick bodies	Frontal lobe, medial temporal lobe, basal ganglia	Broad range of personality changes prior to cognitive decline
	Chronic traumatic encephalopathy (CTE)—sometimes classified as a secondary tauopathy	3R/4R	NFT’s and glial tangles	Frontal and temporal cortices, hippocampus	Personality and behavioral changes, memory loss, and speech and gait difficulty with repetitive trauma history
	Argyrophilic grain disease (AGD)	4R	Argyrophilic grains	Entorhinal cortex, hippocampus, amygdala	Cognitive decline, seizures, personality changes
	Primary age-related tauopathy (PART)	3R/4R	NFTs	Medial temporal mode, basal forebrain, brain stem	MCI or amnestic decline
Secondary	Alzheimer’s disease (AD)	3R/4R	NFTs	Medial temporal lobe, temporal cortex, neocortex	Cognitive decline, changes in behavior, mood swings, language difficulties
	Down’s syndrome	3R/4R	NFTs	Brain stem, cerebellum, frontal, and temporal lobes	In adults—similar to AD patients

NFTs composed of tau are a hallmark of AD. Pathological forms of tau including p-tau closely correlate with changes in cognition [9]. Isoforms of p-tau drive the aggregation of tau forming paired helical filaments (PHF) which make up the main components of the aggregated filaments found in NFTs [12].

The large number of individuals affected by tauopathies and the marked loss of function produced by these disorders have motivated the search for disease-modifying therapies targeting tau pathology. The increasingly well-understood biology of tau, the formation of NFTs, and the cell-to-cell transmission of tau have provided a variety of promising avenues of drug development. Many current drug development programs focus on treating tau pathology in PSP, CBD, PiD, CTE, and FTDP-17 [51, 91, 98]. The search for disease-modifying therapeutics has been pursued most aggressively in AD where therapies have focused on the reduction of amyloid, control of neuroinflammation, and supporting synaptic plasticity [26, 79]. These approaches have led to limited clinical success with two disease-modifying therapies recently approved [43, 56]. Anti-amyloid drugs represent important progress in drug development for AD, but much remains to be done. Interrogation of other potential targets, developing drugs for larger segments of the AD population, and addressing neurodegeneration in non-AD disorders are key next steps in drug development for NDs. Tau-directed therapeutics represent key aspects of these drug development programs.

This review aims to provide information regarding the present and future pipeline of anti-tau therapeutics seeking to treat patients suffering from a variety of NDs involving tau pathology. Key features of the review include the different stages of development of the potential therapeutic drugs as well as the mechanisms of action, molecule types, routes of administration, and recent successes and failures within the tauopathy therapeutic area. The remit of this review is not intended to cover the function, structure, or pathological role of tau. For recent reviews on these topics and extensive discussions on the role of tau as a biomarker, please refer to the references listed here [14, 45, 47, 51, 98]. In this review, we provide a detailed classification of mechanisms of action of agents targeting the modulation of tau (Supplementary Table 1) as well as exploring the emerging science shaping future therapeutic research.

## Methods

Curated search results from targeted queries, including keyword searches for microtubule-associated protein tau (MAPT) and tau, were conducted on GlobalData and SciFinder® databases as the principal sources of information for this therapeutics landscape review. Individual search queries were investigated to reduce false hits and duplications in the database. All filtered results were divided into the developmental stage appropriate to its status. When necessary, additional information was retrieved from company websites, Alzforum, PubMed, clinicaltrials.gov, conference proceedings, or direct communication with company or institute investigators leading the programs. The index date for this review is February 28, 2023, and the tables, figures, and text apply to the information available on that date. We included trials of drugs in clinical trial phases I, II, and III, as well as drugs in preclinical development and in the discovery phase. We acknowledge that many programs in preclinical and discovery phases, especially in academic research centers, have not been publicly disclosed and, therefore, are not included in this review. Tauopathies harbor many types of neuropathology such as inflammation, synaptic dysfunction, oxidative injury, and others that may be indirectly related to the aggregated tau protein but are not themselves an aspect of tau biology. Clinical trials and drug development may target these aspects of pathology within tauopathies. Drugs addressing these downstream aspects of tauopathies may have been omitted or are addressed in less detail in this review.

## Results

### Overview of targets and mechanisms

The majority of therapeutic approaches focus on tau by directly targeting MAPT although indirect mechanisms also afford strategies that may ultimately affect tau pathology [84]. Supplementary Table 1 provides a detailed classification of mechanisms targeting the modulation of tau represented by drugs currently in the tau-directed drug development pipeline.

### Overview of anti-tau drugs in development and their development stages

**Fig. 1:**
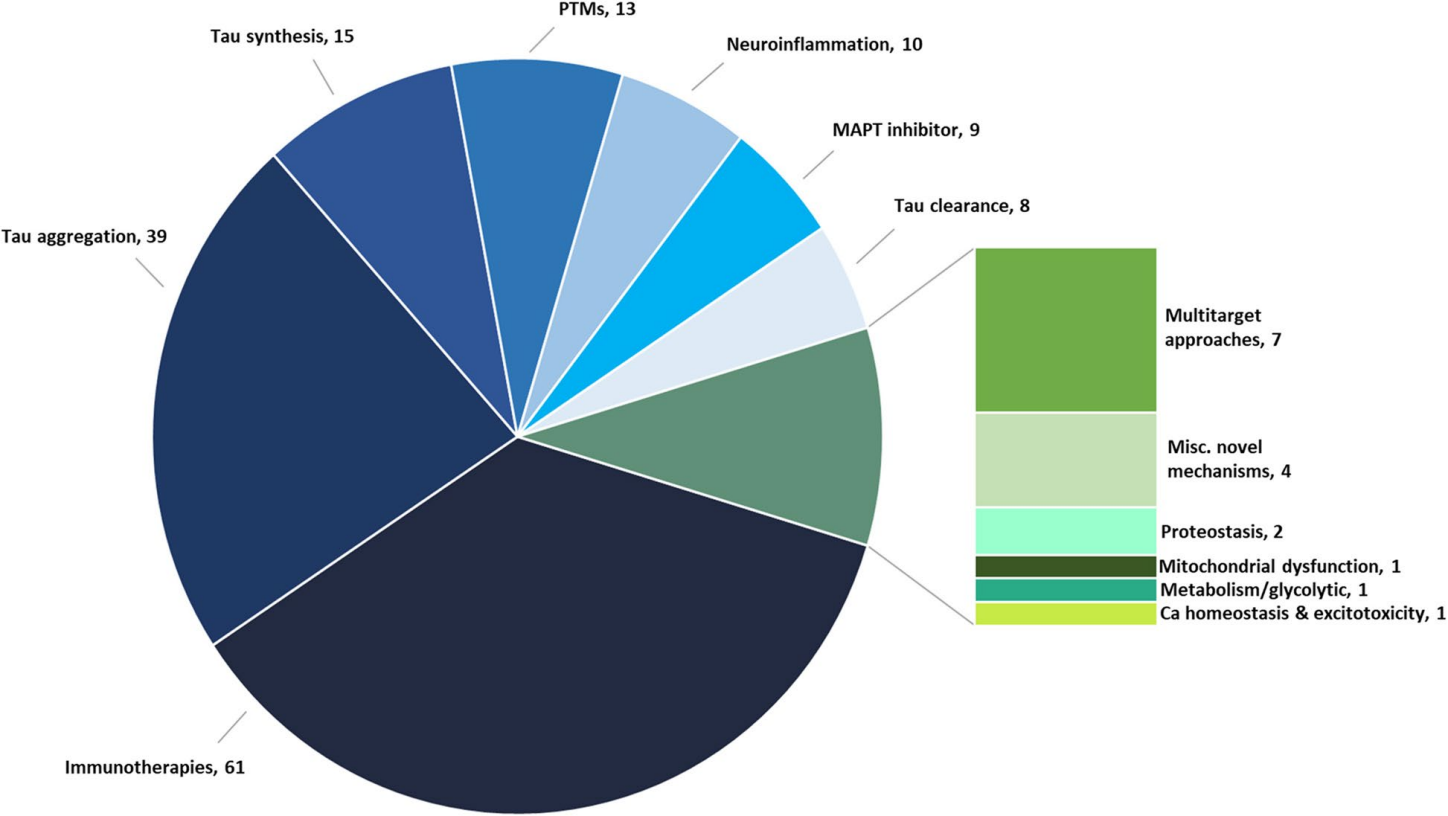
Mechanisms of action of therapeutic programs targeting tau

Our search through the end of February 2023 showed that the tau landscape contains 171 therapeutics, of which 38 are in current clinical development. In Fig. 1, the proportion of projects in discovery and development targeting the principal mechanisms is shown. There is a dominance of immunotherapies (36%) mostly targeting extracellular tau, followed by drugs targeting aggregation (23%), tau synthesis (9%), post-translational modification (8%), neuroinflammation (6%), MAPT inhibitors (5%), tau clearance (5%), proteostasis (1%), and other mechanisms (e.g., mitochondrial dysfunction, metabolism/glycolytic pathways, and calcium homeostasis and excitotoxicity) which each account for less than 1% of the total. Of the remaining drugs, 4% act on multitarget approaches and 2% act on novel mechanisms that have not been fully revealed. They act through a variety of targets that indirectly affect tau by acting on associated pathways which interact with tau (Supplementary Fig. 1).

**Fig. 2:**
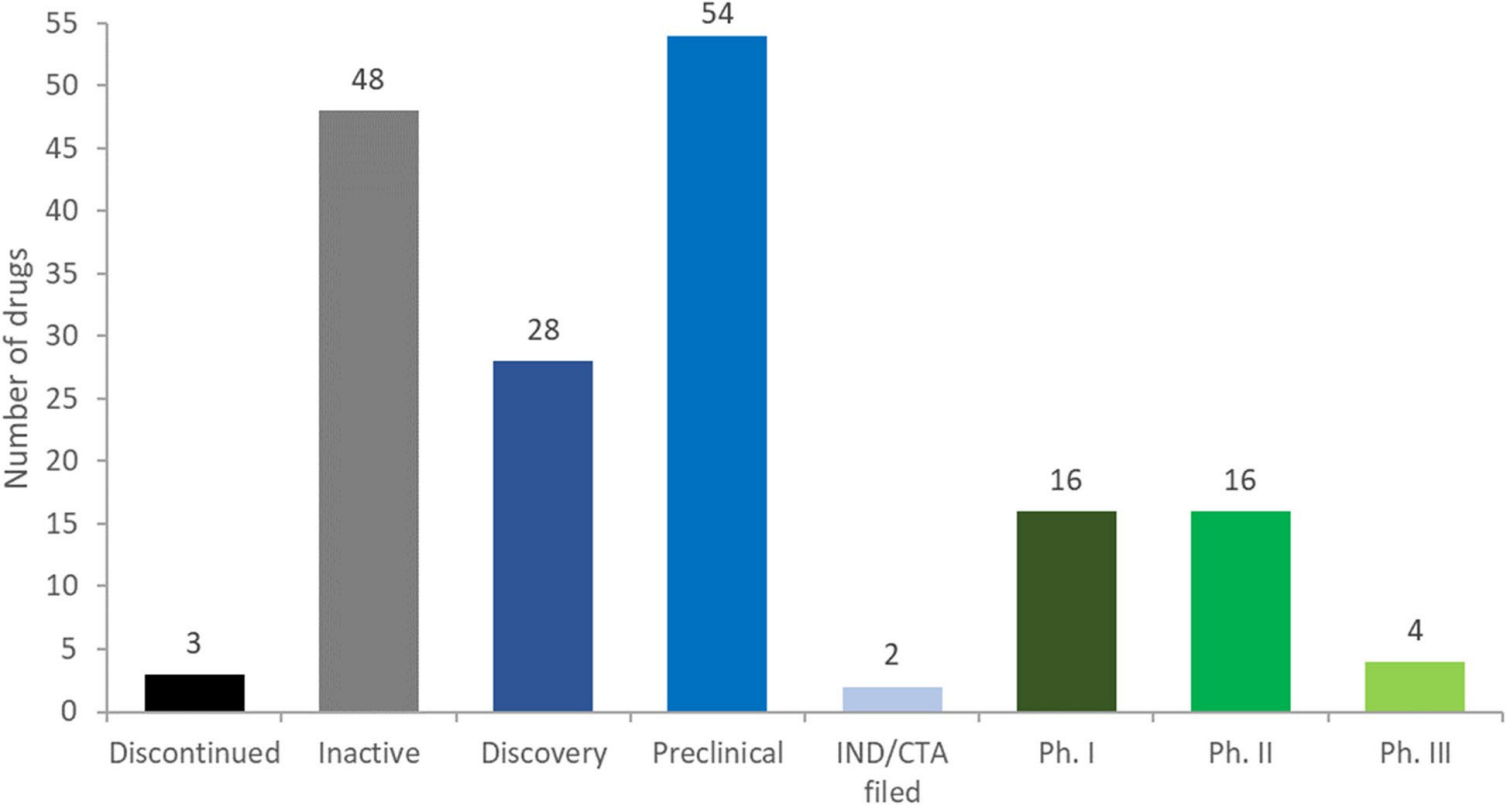
Tau therapeutic portfolio by current development stage

There are no approved drugs that directly target tau. Most anti-tau drugs are in preclinical (31%) or discovery stages (16%) (Fig. 2). Twenty-three percent of the agents discovered in our review are currently active in clinical trials (phases I, II, and III) and two have filed for investigational new drug (IND)/clinical trial application (CTA). There are very few drug candidates in later clinical phases, with only four therapeutics in phase III and 16 are in phase II. There are many “inactive” programs (28%), defined as drugs which have not been updated in more than 2 years if in clinical development, or 4 years if in preclinical development. Of the 38 drugs that are in a clinical trial or which an IND/CTA has been filed, 31 are classified as having a mechanism that inhibits tau, while the remaining drugs are classified as tau agonists (3), tau antagonist (1), neuroprotectant (1), or vaccine (1) or are undisclosed (1) (Supplementary Fig. 2).

### Overview of the types of molecules and routes of administration

**Fig. 3:**
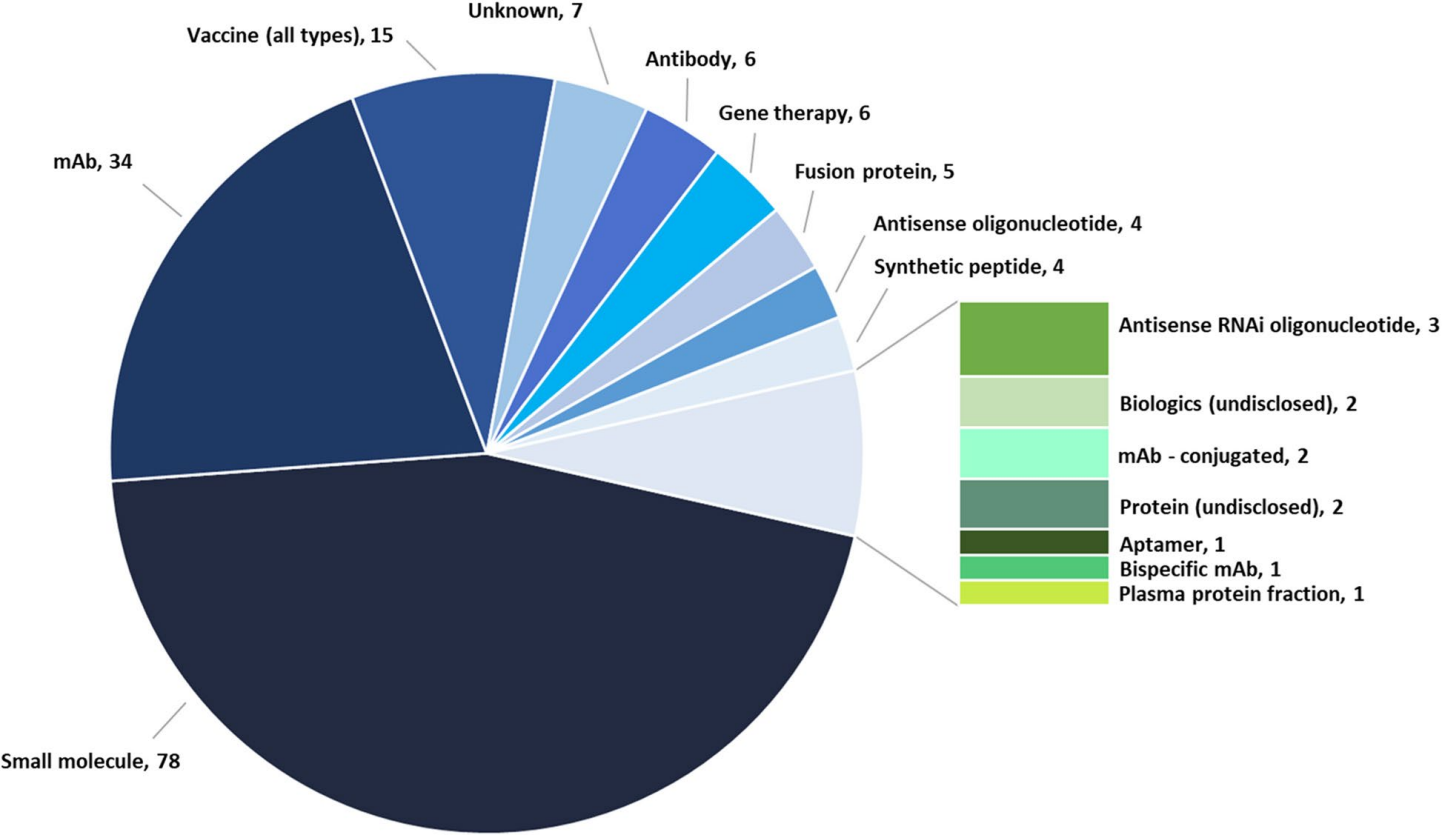
Tau therapeutic portfolio by type of molecule

A growing variety of modalities are being explored in tau therapeutics (Fig. 3), with small molecules accounting for approximately 46% of all therapeutics in development. Monoclonal antibodies (mAbs) constitute a very active and growing area of research representing 20% of therapies in development. In addition, there is an increasing, albeit a still small percentage (< 10% each) of other approaches, including passive and active vaccines, gene therapies, antisense oligonucleotides (ASOs), and therapeutic proteins.

Information on the route of administration for drugs in the clinic (Supplementary Fig. 3) is not always available. Of the 38 drugs in clinical trials, a high proportion of those that are undisclosed are expected to be intravenous (IV) or subcutaneous (SC), reflecting the anticipated route of administration of biologics/antibodies. About 47% of all clinical therapies are reported to be administered orally (PO) as most small molecules are traditionally administered in this way. ASOs are administered intrathecally (IT).

### Tau therapeutic pipeline

This section examines the tau pipeline in detail, highlighting the different approaches currently in discovery and development with most of these drug candidates being labeled as disease-modifying therapies. The landscape of treatments currently in clinical phases is dominated by small molecule approaches and antibodies. Most antibodies focus on mid-domain and p-tau epitopes rather than the N-terminal. In addition to antibody modalities, there are several tau vaccine approaches in the clinic.

#### Phase III

There are currently four therapies, one mAb, and three small molecules, in phase III trials (Table 2). The tau aggregation inhibitor, TRx0237 (HMTM/LMTX/LMTM/methylthioninium chloride), is being developed by TauRx Therapeutics Ltd, for AD dementia and Mild Cognitive Impairment (MCI). It is an orally administered small molecule inhibiting tau and TDP-43 aggregation and dissolves tau filaments in laboratory settings. TRx0237 is currently being evaluated in the LUCIDITY phase III trial. Interim results at the end of the 12-month double-blind trial showed no difference between the treatment (16 mg/day) and placebo groups in the primary endpoints (change in the AD Assessment Scale—cognitive subscale (ADAS-COG_11_), or AD Cooperative Study—Activities of Daily Living scale (ADCS-ADL_23_)) although the rate of decline was less than expected. This trial, unlike a previous phase III study of LMTX, included MCI patients with a positive amyloid positron emission tomography (PET) and excluded patients on concomitant symptomatic treatment. A complicating factor in the trial was the urine discoloration caused by the compound, which can unblind the treatment cohort. A low dose (8 mg/week) of methythionium chloride, a related compound, was administered to the placebo group to color the urine and keep the treatment blinded; unexpectedly, low but possibly therapeutic drug levels were found in the plasma, potentially confounding the trial outcomes. The safety profile is encouraging although the full dataset has not been presented; the 12-month open-label trial extension is ongoing through mid-2023. A previous trial of this agent in FTD demonstrated no difference in the primary outcome comparing the 8 mg and the 200 mg daily dose. Exploratory analyses suggested effects on clinical and magnetic resonance imaging (MRI) measures at the 8 mg dose [82].

**Table 2:** Tau therapeutics in phase III (as of February 28, 2023)

**Molecule Type**	**Company Name**	**Drug Name**	**Indication**	**Route of Administration**	**Mechanism of Action**
mAb	Eisai Co	E-2814 (anti-MTBR tau antibody; BAN 2401)	AD, dementia associated with AD	Intravenous	A humanized, high affinity, IgG1 antibody recognizing the tau MTBR. E2814 and its murine precursor, 7G6, as revealed by epitope mapping, are antibodies bi-epitopic for 4R and mono-epitopic for 3R tau isoforms because they bind to the sequence motif HVPGG.
Small molecule	Annovis Bio	Buntanetap (ANVS-401; Posiphen)	AD, PD	Oral	Inhibits the production of neurotoxic proteins that are derived from APP and tau. It works by inhibiting ⍺-syn, tau and APP synthesis.
	BioVie Inc	NE-3107 (Triolex, HE3286)	AD	Oral	Inhibits extracellular signal-regulated kinase 1 and 2 (ERK 1 & 2). It inhibits the activity of major inflammatory mediators ERK, NFKappaB, and TNF.
	TauRx Therapeutics	LMTX (hydromethylthionine mesylate, TRx 237, HMTM)	AD, MCI	Oral	Tau and TDP-43 protein aggregation inhibitor.

There is limited information available for three of the candidates. Eisai Co.’s mAb E-2814 is a humanized, high-affinity IgG1 antibody targeting tau in AD [18]. It is currently being assessed in the Dominantly Inherited Alzheimer Network Treatment Unit (DIAN-TU). It binds to the HYPGG epitope in the microtubule-binding domain and binds to normal tau as well as NFTs in AD, tau filaments in PSP, and Pick bodies in Pick’s disease [75]. Annovis’ small molecule buntanetap targets ⍺-syn, tau, and amyloid precursor protein (APP) synthesis and is being developed for the treatment of AD and PD patients [38]. BioVie’s small molecule, NE-3107, inhibits the activity of inflammatory mediators for AD patients. A direct link to tau biology is unclear for NE-3107, but it is included in this review for completeness since the reduction of pTau levels was reported in a clinical study [7].

#### Phase II

There are 16 drugs currently in phase II (Table 3). The distribution of approaches favors small molecules with nine in phase II trials. Of all the phase II drugs, only Asceneuron’s small molecule inhibitor is exclusively targeting a primary tauopathy, PSP, while UCB’s mAb, bepranemab, is being used to treat AD (phase II) and PSP (phase I). All other drugs are directed towards the treatment of AD, dementia associated with AD, or general “tauopathies”.

**Table 3:** Tau therapeutics in phase II (as of February 28, 2023)

**Molecule type**	**Company name**	**Drug name**	**Indication**	**Route of administration**	**Mechanism of action**
ASO	Biogen	BIIB 080 (IONIS-MAPTRx)	AD, dementia associated with AD	Intrathecal	ASO acts by targeting tau/MAPT.
mAb	Genentech	Semorinemab	AD	IV, parenteral	Anti-tau mAb acts by targeting misfolded tau.
	Johnson & Johnson	JNJ-3657 (JNJ-63733657)	AD, dementia	IV	A mAb that targets p-tau.
	UCB Biopharma	Bepranemab (UCB-0107, RG 6416)	PSP, AD, tauopathies	IV	Recombinant humanized IgG4P.
Plasma protein fraction	Alkahest	GRF-6019 (ALK 6019)	AD	IV	Proprietary plasma fractions replenish the positive functional chronokines that decrease with age.
Protein	INmune Bio	Pegipanermin (XPro™; XPro1595; DN-TNF; INB 03)	AD	Subcutaneous	TNFR1 antagonist, prevents differentiation and function of myeloid-derived suppressor cells.
Small molecule	Asceneuron	ASN120290 (ASN-561, ASN90)	PSP	Oral	Protein O-GlcNAcase inhibitor.
	EIP Pharma	Neflamapimod (VX 745)	AD	Oral	Inhibitor of the alpha isoform of the protein enzyme p38 mitogen-activated protein kinase (p38 MAPK alpha).
	Eli Lilly and Co	LY-3372689	AD	Oral	Protein O-GlcNAcase inhibitor.
	Oryzon Genomics SA	Vafidemstat (ORY-2001, CNS optimized LSD1 inhibitor)	AD	Oral	Inhibitor of monoamine oxidase type B (MAO-B) and lysine-specific demethylase 1 (LSD1, KDM1A).
	PharmatrophiX	LM-11A31BHS	AD	Oral	Blocks the interaction between beta-amyloid peptide and p75NTR, down-regulating the signaling pathways and up-regulating survival signaling.
	reMYND	REM-0046127 (ReS-19 T)	AD	Oral	Restores calcium dyshomeostasis.
	T3D Therapeutics	T3D-959	AD	Oral	Dual agonist of the peroxisome proliferator-activated nuclear receptor delta/gamma, aka PPARδ/γ (metabolism/glycolytic pathways).
	Univ. of California, Irvine	Nicotinamide	AD	Oral	Histone deacetylase inhibitor to reduce tau-induced microtubule depolymerization and tau phosphorylation.
	Vivoryon Therapeutics N.V	Varoglutamstat (PQ912)	AD	Oral	Blocks the enzyme glutaminyl cyclase (QPCT), which catalyzes the formation of N3pE amyloid. In addition, QPCTL, an isoform of QPCT, is also required for the stability and full potency of the proinflammatory protein CCL2, reducing neuroinflammation. CCL2 is also a promoter of tau pathology.
Vaccine	AC Immune SA (in collaboration with Janssen)	JACI-35054; ACI-35030 (ACI-35, Anti-p-tau vaccine; liposomal anti-p-tau vaccine)	AD, tauopathies	Parenteral	Immune stimulator. Liposomal vaccine based on SupraAntigen technology platform administered through parenteral route. Targets p-tau.

Among the biologics is an ASO by Biogen (BIIB 080) that directly targets tau [66, 76]. An early phase trial demonstrated dose-dependent and sustained reduction of CSF tau and p-tau 181. And unlike the tau immunotherapy trials which also reduced CSF tau levels, these tau ASO-driven changes in CSF tau biomarkers also were accompanied by significant decreases in MK6240 tau PET signal across multiple regions. These results need to be replicated in a larger clinical trial to determine if these central biomarker changes can be replicated and if they are linked to positive clinical outcomes. There are three mAbs sponsored respectively by Genentech, Johnson & Johnson, and UCB. The mAb sponsored by UCB is a recombinant humanized IgG4P. The Genentech mAb targets misfolded tau while the mAb sponsored by Johnson & Johnson targets p-tau. The plasma protein fraction championed by Alkahest aims to replenish the positive functional chronokines which decrease with age. An undefined protein therapeutic by Immune Bio is a tumor necrosis factor receptor 1 (TNFR1) antagonist. The liposomal vaccine in development by AC Immune in collaboration with Janssen is an immune stimulator that targets p-tau.

All nine small molecules in phase II are orally delivered. Asceneuron and Eli Lilly have compounds that are O-GlcNAcase inhibitors, and EIP Pharma is developing an inhibitor of the alpha isoform of enzyme p38 mitogen-activated protein kinase (MAPK). Oryzon Genomics’ inhibitor targets monoamine oxidase type B and lysine-specific demethylase 1. PharmatrophiX is developing a compound that blocks the interaction between Aβ and p75 neurotrophin receptor with effects on Aβ and tau pathology [94]. The compound by reMYND aims to restore calcium dyshomeostasis and exhibits preclinical effects on tau. T3D Therapeutics is advancing a small molecule agonist of peroxisome proliferator activated nuclear receptor delta/gamma. Nicotinamide is being sponsored by the University of California, Irvine, to inhibit histone deacetylase posited to result in reduction of tau-induced microtubule depolymerization and tau phosphorylation. Finally, Vivoryon Therapeutics has a small molecule inhibitor of the enzyme glutaminyl cyclase which is implicated in neuroinflammation and may affect tau biology.

#### Phase I

There are 16 drugs currently in phase I (Table 4). There are also two additional drugs reported to be in the IND/CTA process: first, Dadang & BIO Co.’s (now PharmacoBio) tau and APP aggregation inhibitor, and second, Vitruvian Biomedical’s DNA vaccine. Of the drugs in phase I trials, nine are small molecule approaches, five are mAbs, one is an ASO, and one is a fusion protein. Novartis’ ASO aims to reduce tau and is exclusively targeted at a primary tauopathy, PSP, with no other indication. A fusion protein developed by Proclara Biosciences is designed to block Aβ and tau aggregation; however, there has been no recent activity reported for this molecule. There are five mAbs sponsored respectively by Aprinoia Therapeutics, H. Lunbeck, Prothena, and Merck (2 agents). All the mAbs act to inhibit some form of tau.

**Table 4:** Tau therapeutics in phase I or reported as being in filing for IND/CTA (as of February 28, 2023)

**Molecule type**	**Company name**	**Drug name**	**Indication**	**Route of administration**	**Mechanism of action**
ASO	Novartis AG	NIO-752 (NIO 752; NIO752; tau antagonist)	PSP	Intrathecal	ASO that acts by targeting tau/MAPT.
Fusion protein	Proclara Biosciences	NPT-088	AD	IV	Ig fusion protein that acts by targeting APP, ⍺-syn, prion, and tau protein and is developed based on “General Amyloid Interaction Motif” technology. Blocks aggregate formation of Aꞵ and tau.
mAb	Aprinoia Therapeutics	APNmAb-005 (APN 005)	AD, FTD	Undisclosed	Acts as a tau protein inhibitor and is claimed to have selectivity to toxic tau species.
	H. Lundbeck AS	LuAF-87908	AD, tauopathies	IV	Humanized mouse IgG1 mAb to p-tau protein.
	Merck & Co	MK-2214	AD	Undisclosed	An anti-tau mAb.
		Ta-1505	AD	Undisclosed	Inhibits pSer413-tau to inhibit excessive phosphorylation of tau.
	Prothena	PRX-005	Phase I for AD; preclinical for CTE, FTD, PSP	Subcutaneous	The mAb functions as MAPT inhibitor.
Small molecule	Alterity Therapeutics	AHT-434 (PBT-434)	MSA (Ph. II); CBD, PD, PSP (Ph. I in Australia only)	Oral	Inhibitor of metal-protein interaction. Prevents cell death by inhibiting the interaction between dopamine and iron and stops the accumulation of ⍺-syn (possibly tau too). It also elevates levels of the protective protein called DJ-1, reducing the rise of oxidative stress.
	Anavex Life Sciences	ANAVEX 3–71 (AF-710B)	AD, FTD, PD	Oral	Induces a synchronized sigma-1 receptor activation and M1 muscarinic allosteric/bi-topic modulation via super-sensitization of M1mAChR, through a hypothetical heteromerization with Sig1R. Decreases Aꞵ, tau-hyperphosphorylation, GSK3beta activation, and prevents apoptosis and mitochondrial dysfunction via increased Bcl2.
	BeyondBio Inc	BEY-2153	AD	Oral	Acts as a tau and Aꞵ protein inhibitor.
	Biogen Inc	BIIB-113	AD	Oral	Protein O-GlcNAcase inhibitor.
	Cortice Biosciences	ARC-100 (TPI 287)	AD, PSP, CBD	IV, oral	Microtubule inhibitor. Binds to tubulin and stabilizes microtubules, resulting in inhibition of microtubule assembly/disassembly dynamics, cell cycle arrest at the G2/M phase, and apoptosis.
	Eli Lilly and Co	ACI-3024 (morphomer tau aggregation inhibitors)	AD, tauopathies	Oral	Reduction of tau aggregation. Selective binding to tau, no binding to the monomeric form of tau, and selective binding to AD brain-derived pathological tau.
	Neurokine Therapeutics	MW-150	AD, dementia, tauopathies	Oral	General mechanism for reducing neuroinflammation through selective inhibition of mitogen-activated protein kinase p38 (p38MAPK).
	Oligomerix Inc	OLX-07010 (TO-0582)	AD, FTD, tauopathies	Undisclosed	Acts by targeting tau oligomer formation.
	Revivo Therapeutics	RIV-5061 (RIV-1061)	AD; cognitive impairment in IND/CTA phase	Undisclosed	Immediate release formulation of nomethiazole. It acts by targeting APP and NFTs. The drug candidate works by inhibiting and reducing tau protein from forming PHFs.
Unknown	PharmacoBio (formerly Dadang & BIO Co. Ltd.)	DDNA-0101	Dementia associated with AD (in IND/CTA)	Undisclosed	Aggregation inhibitor for tau and APP. Also prevents the hydrolysis of acetylcholine and enhances cholinergic function. Developed based on gut-brain microbiota axis platform.
Vaccine	Vitruvian Biomedical	YM-7555, reported to be in IND/CTA filed phase	AD (in IND/CTA)	Undisclosed	DNA vaccine that comprises human Aꞵ 1–42 and human tau 379–408 sequences connected to both ends of the Fc portion of immunoglobulin.

Various mechanisms of action are covered among the nine small molecules being assessed in phase I. Alterity Therapeutics’ small molecule candidate inhibits metal-protein interaction and is in phase I (Australia) for CBD, PD, and PSP. It is also in phase II (US) for the synucleinopathy and multiple system atrophy (MSA). Anavex Life Sciences’ small molecule activates sigma-1 receptor and modulates M1 muscarinic allosteric/bitopic to decrease tau hyperphosphorylation. BeyondBio is testing a tau and Aβ inhibitor, and Biogen is testing an O-GlcNAcase inhibitor. Cortice Biosciences’ microtubule inhibitor is being tested via IV and oral formulation. Eli Lilly is testing an orally administered small molecule which reduces tau aggregation by selectively binding to pathologic forms of tau while not interacting with monomeric tau.

Neurokine Therapeutics is developing a small molecule that reduces neuroinflammation-related tau production by inhibiting mitogen-activated protein kinase p38. Oligomerix recently started enrollment for their approach aimed at eliminating tau oligomer formation. Revivo Therapeutics has not disclosed the route of administration of its drug, but it is noted to be of an immediate-release formulation of nomethiazole which targets APP and NFTs and is posited to prevent tau from forming PHFs.

#### Discovery and preclinical development

This section considers all tau treatment development programs that have not yet entered clinical stage testing. There are 54 drugs classed as being in the preclinical stage of development (Supplementary Table 2). The definition of preclinical is the stage at which a drug is tested in non-human species and in vivo studies for the purpose of understanding the efficacy, toxicity, and pharmacokinetics of the candidate drug. Safety in animals predicting safe use in humans must be shown in this stage before the drug can be progressed to clinical testing. The distribution of drug types and quantity (#) in the preclinical phase of development are as follows: antibody (2), antisense RNAi oligonucleotide (2), ASO (2), bispecific mAb (1), DNA vaccine (1), gene therapy (4), mAb (11), small molecule (20), subunit vaccine (5), synthetic peptide (2), vaccine (2), and unknown (2).

There are 28 drug candidates considered to be in discovery (Supplementary Table 3). The discovery phase terminology is used when the project is in the process of identification and optimization of a substance for therapeutic use with the aim of producing a candidate for preclinical testing. Candidates are primarily identified through the assay of compounds against biological targets. Positive hits are screened for other key characteristics such as bioavailability, toxicity, and potency and optimized through drug design processes. Once a candidate drug is elected and is to be tested in more complex biological systems, it is advanced to the preclinical stage. This stage begins once a lead candidate or small estate of promising candidates have been identified. The distribution of drug types and quantity (#) in the discovery phase of development are as follows: antibody (2), aptamer (1), conjugate vaccine (2), fusion protein (3), gene therapy (1), mAb (5), small molecule (12), and unknown (2).

#### Inactive and recently discontinued programs

There are 48 programs classified as “inactive” which we define as a drug which has not been publicly updated in more than 2 years if in clinical development, or 4 years if in preclinical development. Although the information on these drugs (Supplementary Table 4) might not be exhaustive due to the lack of information, it is important to consider the volume of research they comprise across a variety of mechanisms. The distribution of drug types and quantity (#) that have been in development but are currently inactive are as follows: antibody (2), antisense RNAi oligonucleotide (1), biologic (2), fusion protein (1), gene therapy (1), mAb (8), recombinant protein (1), small molecule (25), subunit vaccine (1), synthetic peptide (2), vaccine (2), and unknown (2).

A few drug candidates have been discontinued due to a lack of efficacy in the clinic. Among these clinical failures, the anti-tau antibody therapeutics in Supplementary Table 5 have had the most significant impact on both tauopathy and AD development programs. Tilavonemab, (ABBV-8E12), a recombinant monoclonal antibody that recognizes the N-terminal of misfolded extracellular aggregated tau, a form of tau that has been implicated in the seeding and transneuronal propagation of pathological tau, was being developed by AbbVie and C2N Therapeutics in AD and PSP [92]. The development of tilanonemab was halted in 2021 after it failed to show effects on primary and secondary outcomes in a phase II clinical trial on AD patients with confirmed AD-positive amyloid positron emission tomography (PET) [46]. The drug did not halt brain atrophy or decrease neurofilament light (NfL) in plasma. Tilavonemab’s development in PSP was halted after it failed to show efficacy over placebo even though target engagement had been established by a demonstratable decrease of free tau in cerebrospinal fluid (CSF).

Gosuranemab (BIIB092, BMS-986168) is another monoclonal antibody that recognizes N-terminal forms of aggregated extracellular tau [27]. This therapeutic advanced to phase 2 studies in PSP and AD and was part of a basket trial in primary tauopathies. The antibody showed a dose-dependent accumulation in serum and CSF, and the N-terminal forms of tau in CSF were reduced by more than 90% for all doses. Gosuranemab showed no efficacy in primary and secondary outcome measures compared to the placebo. Biogen halted all development efforts in AD, PSP, and other primary tauopathies.

Zagotenemab (LY3303560), a humanized tau mAb being developed by Eli Lilly, was discontinued from further development [1, 65]. This antibody was originally developed by Peter Davies from MCI-1, an agent binding an N-terminal epitope present in an early pathological conformational form of tau [54]. In preclinical studies, zagotenemab reduced levels of insoluble p-tau and NFT pathology. However, in the phase II trials conducted in North America and Japan, this agent failed to meet its primary endpoint [33].

TPI-287 was a microtubule stabilizer assessed in a basket trial including patients with AD, PSP, and CBD. No efficacy was observed and blood–brain concentrations may have been less than anticipated. The treatment led to anaphylactoid reactions in three AD patients treated with drug compared to placebo; PSP patients on active treatment had more falls than those on placebo; and PSP and CBD patients exhibited cognitive decline [88]. Development has been terminated.

## Discussion

Cellular inclusions (NFTs) and “miliary foci” (amyloid plaques) were recognized in the original neuropathological studies conducted by Alois Alzheimer and reported in 1907 for the disease now known by his name. In 1989, the intracellular tangles of AD were shown to be composed of p-tau that assumed a double helical configuration within the cell [52]. Autopsy studies of patients assessed in life demonstrated a stronger relationship between NFT burden and cognitive decline than between amyloid plaque burden and cognition [3, 69]. Braak and Braak investigated the relationship of cognition to NFT pathology across the spectrum of symptomatic AD and observed a systematic progression of stages from the entorhinal cortex (stages I and II), to limbic cortex (stages III and IV), and to neocortical regions (stages V and VI) [9]. The progressive neurogeographical involvement of the brain by tau pathology has been related to a prion-like spread from neuron to neuron of tau fibrils and other types of tau fragments [31]. The relationship between NFT pathology and cognition has been confirmed by recent studies showing the correlation between abnormalities visualized on tau PET and cognitive decline [4, 28]. Studies of non-AD tauopathies demonstrate that the tau of each tau-related disorder (CBD, PSP, CTE, PiD) has unique structural features [36, 37, 97]. Cell-to-cell contagion of tau similar to that observed in AD has been shown in non-AD tauopathies [41]. In tauopathies, the protein undergoes post-translational modification (PTM) including phosphorylation, acetylation, glycation, and ubiquitination. The PTMs impair the ability of tau to function normally in microtubule organization and stability. P-tau aggregates into oligomers that exhibit substantial neuronal toxicity before fibrillizing to form NFTs [73]. P-tau contributes to synaptic dysfunction, mitochondrial impairment, inflammation, and neurodegeneration [68]. These and many other biological and mechanistic observations have provided the basis for insight into AD-related and non-AD tauopathies and have guided treatment hypotheses [14].

The complexity of tau biology and pathological alterations of tau in the tauopathies is reflected in the diversity of therapeutic approaches evident in this review. Of the 171 drugs (including those that have been discontinued or are inactive), 61 are tau-related immunotherapies, 39 target tau aggregation, 15 are directed at tau synthesis, 13 are focused on PTMs, 9 are MAPT inhibitors, and 8 target tau clearance (Fig. 1). The most common nonimmunologic mechanisms of agents in the pipeline are inhibition of tau production, phosphorylation, aggregation, or toxicity (Tables 3 and 4). A few drugs are stimulators or activators whose mechanistic goal is to increase the clearance of tau proteins through a variety of cellular actions including autophagy. In addition to the drugs targeting tau mechanisms directly, there are a variety of tau-related pathologies for which therapies are being developed for use in tauopathies including drugs directed at neuroinflammation, mitochondrial dysfunction, metabolism and bioenergetics, and calcium homeostasis and excitotoxicity (Fig. 1). These latter mechanisms occur in other NDs and potential therapeutic translation across conditions will depend on the disease-based uniqueness of the relationship of the tau changes to the secondary mechanism.

The progressive stages of pathologic tau formation are conceptualized as transcription to MAPT; translation and post-translational changes of tau to integrate into the microtubule; generation of dimers, oligomers, and protofibrils; and formation of filaments and NFTs. Transynaptic prion-like propagation of tau may involve dimers, protofibrils, tau-bearing exomes, or aberrant forms of tau [59]. As shown in Fig. 1, the most common target among pipeline drugs directed at tau biology is the transynaptic extra-neuronal phase addressed by immunotherapies, followed by tau aggregation, tau synthesis, post-translational modifications of tau, and clearance of tau. Therapeutic targets within the domain of PTMs and targeted by drugs in the pipeline include tau phosphorylation, tau acetylation, tau glycosylation (O-GlcNAcase inhibitors), and tau truncation [91].

Only 36 of the 171 drugs reviewed are currently in clinical trials, and only four are in phase III. There are 16 drugs in phase II and 16 in phase I. The outcomes of these trials will be highly informative regarding promising targets, use of biomarkers, appropriate populations, and trial design and conduct. The high proportion of drugs in preclinical (*N* = 54) and discovery (*N* = 28) stages of development suggest that the growing understanding of tau biology is stimulating laboratory studies of possible therapeutic candidates. The progress of these drugs towards the clinic will provide learnings concerning animal models of tauopathies, use of induced pluripotent stem cells in therapeutic development, challenges to defining pharmacokinetic and pharmacodynamic responses in the preclinical setting of tau therapy development, and features of molecules that translate into clinical application [42, 44]. The outcome of trials will inform preclinical development strategies through reverse translation approaches [80].

Prion-like cell-to-cell transfer of tau fibrils that serve as templates for tau pathology in the receiving neuron and promote further seeding of tau provides the basis for immunotherapies directed at extracellular tau in AD and tauopathies. The period of extracellular residence of tau is the only chapter in the tau life cycle not requiring intracellular penetration and vulnerable to these therapies. Several immunotherapy programs for the development of agents directed at various aspects of extracellular tau have been initiated and immunotherapies represent the largest single mechanistic category in the tau pipeline. None of these clinical-stage programs has succeeded, three have been discontinued for lack of efficacy in human trials (Supplementary Table 5), and 48 programs are inactive. Of 16 tau-related drugs in phase II trials, three are mAbs and one is a vaccine. Similarly, of 16 drugs in phase I development, five are mAbs. There are 54 drugs in preclinical development, 14 of these are protein or antibody-based drugs and eight are vaccines. The robust number of preclinical and early-stage clinical programs developing immunotherapies indicates a continuing interest in the extracellular tau targets and optimism regarding vulnerability of the protein during the inter-cellular passage of tau.

There is a tremendous need to discover and develop clinically applicable biomarkers that can be used to understand the cellular and molecular pathways implicated in tauopathy pathophysiology and support the development of mechanism-based therapeutics [24, 77]. Tau biomarkers are becoming increasingly available in AD. Currently, tau PET and CSF and plasma tau and p-tau measures are the dominant biomarkers used to diagnose and study tau biology in AD ([10, 71, 98]). These approaches are being implemented in clinical trials and promise to accelerate drug development [22, 93]. Amyloid PET is used to confirm a diagnosis of AD and document the pharmacodynamic response to anti-amyloid mAb therapy [15]. Documentation of amyloid reduction is accepted by the FDA as reasonably likely to predict clinical benefit and served as a basis for accelerated approval of aducanumab and lecanemab [32].

Progress in the development of fluid biomarkers for tauopathies has been less successful than advancing biomarkers for AD. Available measures are useful in identifying AD which may be difficult to distinguish from tauopathies in some circumstances. For AD, Aꞵ 42/40 ratios and specific p-tau isoforms, such as p-tau181 and p-tau 217, have been useful for diagnosis and have recently shown utility in monitoring and assessing outcomes of amyloid-based clinical trials [48, 96]. However, no fluid biomarkers have shown comparable utility for primary tauopathies. Plasma NfL, a general marker of neuronal degeneration, is being studied as a possible biomarker for disease progression in PSP and frontotemporal lobar degeneration (FTLD), [5, 29] but has shown mixed results as an outcome marker [17, 39, 60, 95]. Plasma NfL was shown to predict clinical decline and to correlate with cortical thickness in FTLD [50]. The ratio of glial fibrillary acid protein (GFAP)/NfL distinguished FTD-tauopathy from FTD due to TDP-43 pathology and has the promise as a biomarker to identify the tau subpopulations of FTD [20]. The recent accelerated approval of toferson by the FDA on the basis of reduction in NfL and the superoxide dismutase 1 (SOD1) protein in patients with ALS resulting from a SOD1 mutation suggests a readiness of some regulatory agencies to accept biomarkers as approvable trial outcomes prior to demonstration of robust clinical benefits [63]. Recently, CSF tau peptides derived from the microtubule-binding region (MTBR) of 4R-tau have shown potential as a fluid biomarker to differentiate some primary tauopathies from controls [49]. Recent modeling of the temporal ordering of biomarker changes suggests a possible framework for integrating biomarker and clinical changes in prevention trials [85]. If confirmed, these biomarkers can be used in drug development and clinical trials to identify patients, monitor disease progression, stratify patient populations, track or predict the therapeutic response, and define pharmacodynamic endpoints [13, 25]. The experience with amyloid PET in AD and NfL in ALS suggests that tau biomarkers might mature to provide sufficient confidence to serve as the basis for accelerated approval and eventually to be surrogate outcomes predictive of clinical benefit [6].

Tau PET enables the visualization of pathological tau aggregates in vivo and plays an increasingly important role in AD clinical trials. It has been used for patient selection and as an outcome to assess the effects of anti-amyloid monoclonal antibodies on tau pathology [11, 64]. Currently, [18F]flortaucipir (Tauvid™) is the only FDA-approved compound used to monitor tau burden in the brains of AD patients [55]. Flortaucipir binds well to the mixed 3R/4R tauopathy of AD but does not perform well in other tauopathies which have disease-specific differences in tau isoforms [2, 87]. Next-generation tau PET ligands may allow visualization of fibrillar tau deposits in non-AD tauopathies ([45, 58, 62, 72, 86]). Within single isoform tauopathies, cryo-electron microscopy studies identify different core structures for the tau filaments complicating the development of tau ligands [81]. The development of tau PET ligands applicable to primary tauopathies has the potential to accelerate tau-related clinical trials and drug development programs.

In summary, the pipeline of treatments for tauopathies—both in the context of AD and non-AD tau pathology—is best populated in discovery and preclinical stages as well as early phase trials. A diverse array of targets is represented in the pipeline with an emphasis on immunotherapies directed at extracellular tau. The necessary repertoire of biomarkers needed to guide drug development is lacking in tauopathies, but progress is being made in both fluid and imaging biomarkers. The integration of new targets with innovative drugs and novel biomarkers promises to accelerate the development of therapies for tau disorders.

### Supplementary Information

**Additional file 1: Supplementary Table 1.** Classification of therapeutic targets for tauopathies. **Supplementary Table 2.** Tau therapeutics in preclinical development (as of February 28, 2023). **Supplementary Table 3.** Tau therapeutics in discovery (as of February 28, 2023). **Supplementary Table 4.** Inactive tau therapeutic programs (as of February 28, 2023). **Supplementary Table 5.** Recently discontinued tau therapeutic programs (as of February 28, 2023). **Supplementary Figure 1.** Therapeutic approaches in development that indirectly target tau.** Supplementary Figure 2. **Mechanism of action of drugs in clinical trials. **Supplementary Figure 3.** Routes of administration for tau therapeutics in clinical trial phase I, II, and III.

## Data Availability

Not applicable.
