# Supplementary material for: The therapeutic landscape of tauopathies: challenges and prospects
Source: Alzheimers Res Ther. 2023 Oct 6;15:168. doi: 10.1186/s13195-023-01321-7 (PMC10557207; doi:10.1186/s13195-023-01321-7)

**Supplementary information for:**

**The Therapeutic Landscape of Tauopathies: Challenges and Prospects**

**Authors:**

Jeffrey L. Cummings,^1^ M. Isabel Gonzalez,^2^ Martyn C. Pritchard,^2^ Patrick C. May,^3^ Leticia M. Toledo-Sherman,^4^ Glenn A. Harris,^5^

**Affiliations:**

1: Chambers-Grundy Center for Transformative Neuroscience, Dept. of Brain Health, School of Integrated Health Sciences, Univ. of Nevada, Las Vegas (UNLV), Henderson, NV, USA

2: Drug Discovery and Development Consultants Ltd., Cambridge, UK

3: Advantage Neuroscience Consulting LLC, Fort Wayne, IN, USA

4: MycRx Pharma, Austin, TX, USA

5: Rainwater Charitable Foundation, Fort Worth, TX, USA

**Correspondence:**

Glenn A. Harris, Rainwater Charitable Foundation, 777 Main Street, Suite 2250, Fort Worth, Texas 76102, email:[gharris@rainwatercf.org](mailto:gharris@rainwatercf.org)

**Supplementary Table 1:** Classification of therapeutic targets for tauopathies

| **Therapeutic Target** | **Therapy Classification** | **Additional Notes and Examples** |
| --- | --- | --- |
| Tau synthesis | Genetic interference | micro-RNAs (miRNAs), ASOs |
|  | Splicing mechanisms | Blockade or enhancement of alternatively spliced exons |
|  | Tau translation | Natural antisense transcripts |
| Tau post-translational modifications (PTMs) and crosstalk | Tau hyperphosphorylation | miRNAs, protein kinase inhibitors, phosphatase activators |
|  | Tau acetylation | Increased degradation of p-tau |
|  | Tau ubiquitination |  |
| Proteostasis | Tau ubiquitination |  |
|  | Tau proteolysis |  |
|  | Stress granules associated RNA binding proteins |  |
| Tau aggregation | Detachment of abnormal tau from microtubules/axonal dysfunction | Increasing microtubule stabilization |
|  | Intrabodies | Binding to intracellular tau to prevent aggregation |
|  | Tau acetylation | Through CBP and P300 |
|  | Stress granules-associated RNA binding proteins |  |
|  | Oligomer formation |  |
|  | NFT formation | Chaperone-mediated folding mechanisms (heat shock protein, Hsp) |
|  | Combined inhibitors of tau and amyloid aggregation |  |
| Promote tau clearance | Tau acetylation | Through CBP and P300 |
|  | Ubiquitin proteasome system | Deubiquitinating enzymes |
|  | Autophagy lysosome system | Chaperone mediated autophagy, microautophagy, macroautophagy |
| Tau propagation | Secretion of tau to extracellular space | Mechanisms affecting receptors, exosomes, or microglia |
|  | Trans-synaptic tau pathway |  |
|  | Entry of tau into neighboring neuron | Mechanisms related to endocytosis or exosomes |
| Neuroinflammation | Microglial uptake of pathological tau |  |
|  | Microglia targets | ApoE, Trem2, Inflammasome - NLRP3 |
|  | Modulation of neuroimmune system |  |
| Mitochondrial dysfunction | Reduction of oxidative stress | KEAP1 - NRF2 |
|  | Improvement of mitochondrial oxidation |  |
|  | Mitochondrial unfolded protein response |  |
|  | Generation of induced microglia |  |
|  | Modulation of mitochondrial translocator protein |  |
| Metabolism/glycolytic pathways | Metal ion homeostasis, ferroptosis |  |
|  | Changes in neurotransmitter, lipid, or energy metabolism |  |
|  | NAD modulators, PARP inhibition |  |
|  | Glycolysis |  |
| Multitarget | Amyloid and tau combined reduction |  |
| Immunotherapies | Active vaccines |  |
|  | Monoclonal antibodies |  |
| Neuronal axonal dysfunction |  |  |
| DNA damage/repair |  |  |
| Ca homeostasis and excitotoxicity | Calcium channel modulators | TRPML1 |
| Trophic support |  |  |
| Brain/gut axis and microbiome | Insulin resistance |  |
|  | CNS cholesterol |  |
| Glymphatic system |  |  |
| Misc. - novel mechanisms and targets |  |  |

**Supplementary Table 2:** Tau therapeutics in preclinical development (as of February 28, 2023).

| **Molecule Type** | **Company Name** | **Drug Name** | **Indication** | **Mechanism of Action** |
| --- | --- | --- | --- | --- |
| Antibody | ICB International | Antibody to inhibit p-tau | AD | Targets p-tau protein. |
|  | ICB International | Antibody to inhibit tau | AD | Targets tau protein. |
| Antisense RNAi oligonucleotide | DTx Pharma | Antisense RNAi oligonucleotides to inhibit MAPT for tauopathies | Tauopathies | Antisense RNAi oligonucleotides that act at the tau protein. |
|  | Eli Lilly and Co. | DCRLLY-11 | NDs | Acts by inhibiting the expression of MAPT. It is based on GALXC RNAi technology. |
| ASO | AcuraStem | AS-202  (5-10-5 MOE gapmer) | FTD, ALS | PIKFYVE kinase inhibition potentially leading to blocking autophagosome-lysosome fusion and exosomal secretion. |
|  | Washington Univ. in St Louis | ASO to inhibit tau protein for NDs | AD, ALS, HD | ASO that acts as a tau protein inhibitor. |
| Bispecific mAb | Denali Therapeutics | ATV-Tau | AD | Bispecific monoclonal antibodies (ATV:Tau bivalent) that act by targeting tau. |
| DNA vaccine | Capo Therapeutics | AV-1980D | AD | DNA vaccine developed based on MultiTEP platform. It exhibits immunostimulant properties, acts by targeting tau protein (N-terminus). |
| Gene therapy | Biogen Inc. | ST-501 (AAV-ZFP-MAPT) | AD, tauopathies | Adeno-associated virus (AAV) vector encoding zinc finger protein transcription factor (ZFP-TF) mediated by gene regulation technology. It acts by inhibiting tau protein. |
|  | Lacerta Therapeutics | Gene therapy to inhibit Aꞵ and tau protein for AD | AD | Target Aβ and tau proteins. The drug candidate is developed based on OneBac AAV vector technology platform. They use a vectorized antibody to target the accumulation of amyloid-beta and tau proteins. |
|  | Voyager Therapeutics | Various tauopathies | AD, dementia, PSP | AAV gene therapy of a vectorized version of an anti-tau mAb. |
|  | Weill Cornell Medical College | Gene therapy to inhibit tau protein for CTE | CTE | Gene therapy delivers mAb that inhibits tau protein. |
| mAb | ADEL | ADELY-01 | Dementia associated with AD | Targets the pathologically modified epitope of tau protein. |
|  | AGP Therapeutics | Armanezumab | Tauopathies | Humanized mAb specific to N-terminus of pathological tau. |
|  | Axon Neuroscience | AADvac-2 | AD, tauopathies | Provides passive immunity which is transferred via active humoral immunity in the form of a mAb. It acts by targeting tau. |
|  | Beth Israel Deaconess Medical Center | mAb for CNS | AD, NDs, TBI | Inhibits cis-tau protein by specifically detecting and eliminating the pathogenic conformations of tau. |
|  | Johnson & Johnson | mAb to inhibit tau for AD (dmCBTAU 22.1) | AD | Antibody CBTAU-22.1 was isolated from the memory B-cell repertoire of healthy human donors. CBTAU-22.1 binds a disease-associated p-tau epitope in the C-terminus (Ser422). |
|  | New York Univ. | mAbs to inhibit tau for CNS disorders | AD, dementia, tauopathies, FTD | mAbs that act as MAPT inhibitors. |
|  | NYU Langone Health System | mAbs to inhibit tau, Aꞵ and ⍺-syn for NDs | AD, NDs | mAbs that act by inhibiting tau protein, Aꞵ peptide and ⍺-syn. |
|  | Pinteon Therapeutics | PNT-001 | AD | Inhibits tau (cis-pT231tau). |
|  | ProMIS Neurosciences | Tau (microtubule stabilization, neurite development) | AD | Dissolves misfolded tau proteins and reduces NFTs to improve memory. |
|  | Synapss Therapeutics | SMQ-1001 | AD, PD | Inhibits ⍺-syn and aggregation of tau directly and also eliminates the ability of cross-seeding between the proteins. |
|  | UCB Biopharma | mAbs to inhibit tau protein for AD and PSP | AD, PD | Decreases insoluble tau and improves motor functions. |
| Small molecule | ADRx Inc. | Small molecules to inhibit MAPT for AD | AD | Tau aggregation inhibitors. |
|  | Anavex Life Sciences | ANAVEX-141 | AD, PD | Prevention of endoplasmic reticulum stress, mitochondrial stress, and oxidative stress. It prevents the expression of caspase 3, an enzyme that plays a key role in apoptosis. |
|  | Aprinoia Therapeutics | Tau degrader | AD, FTD, PSP, tauopathies | Engages pathological tau aggregates at specific binding pockets only present in toxic tau aggregates to the ubiquitin-proteasome system for further degradation. |
|  |  | Tau modulator | AD, FTD, PSP, tauopathies | The compound binds to pathological tau aggregates and modulates their assembly, converting the tau aggregates into harmless conformations. |
|  | Arvinas | Tau | AD, FTD, PSP | The therapeutic candidate is developed based on PROTACs (proteolysis targeting chimeras) technology. It inhibits the misfolded forms of tau and prevents aggregation. |
|  | Augustus BioTarget | Curcumin + paclitaxel | AD | CLR-TargoSphere-encapsulated paclitaxel and curcumin inhibit both tau protein and Aꞵ peptide. Curcumin acts by inducing the degradation of Aꞵ deposits and uptake by macrophages. Paclitaxel inhibits hyperphosphorylation of abnormal tau protein. |
|  | Autotac Bio | ATC-102 | AD | Through the AUTOTAC platform the molecules selectively sequester tau aggregates and target them to the lysosome for autophagic degradation. |
|  | Axon Neuroscience | Small molecules to inhibit tau for AD | AD | Blocks tau-tau oligomerization. |
|  | Biogen Inc. | Compound 31 in paper | AD | TTBK1 Inhibitor. |
|  |  | Small molecule to inhibit tau for AD | AD | Undisclosed |
|  | Cantabio Pharmaceuticals | CB-301 | AD, PD | Small molecule pharmacological chaperones which bind to the native monomeric tau protein and reduce its aggregation. |
|  | Cesa Alliance SA | AA-7 (Relumninol) | AD | Inhibits the production of neurotoxic proteins that are derived from the APP and tau. It works by inhibiting ⍺-syn, tau, and APP synthesis. |
|  | Expansion Therapeutics | Small molecules to inhibit tau for tauopathies ("AD Tau") | Tauopathies | Acts by modulation of splicing of pre-mRNA of MAPT. It leads to nonsense-mediated decay of mRNA and thereby lowers the levels of tau. |
|  | Harvard Univ. | Small molecule to inhibit USP14 for AD and PD | AD | It stimulates proteasome-mediated clearance of the proteins by inhibiting the enzyme USP14. |
|  | reMYND NV | ReS-3T | AD, epilepsy | Targets a central regulator of neuronal activity to restrain neuronal hyperactivity in a disease context. The treatment counters tau and Aꞵ driven toxicity. |
|  | TauRx Therapeutics | Small molecule to inhibit tau for AD | AD | Blocks the formation of tau oligomers and their conversion to PHFs. It also helps to solvate the tau oligomers and PHFs into short, truncated monomers which comprise the proteolytically stable core of the PHFs. |
|  | Tongji Medical College | C-004019 | AD | A PROTAC which targets tau protein degradation. It is designed to simultaneously recruit tau and VHL E3-ligase to selectively enhance ubiquitination and proteolysis of tau. |
|  | Treventis | Small molecules to inhibit Aꞵ and tau protein for AD and tauopathies | AD, tauopathies | Neutralizes misfolded proteins to restore normal functioning. |
|  | Univ. of California Los Angeles | CLR-01 | AD, PD, spinal cord injury | A molecular tweezer which has the capability of binding to other proteins. It targets ⍺-syn, tau and APP. |
|  | Yuma Therapeutics | YT-17 | AD | Acts as Hsp90 inhibitor. Induces production of Hsp70 and Hsp40, as well as of other chaperones, which in turn, promote disaggregation and protein degradation. |
| Subunit vaccine | Capo Therapeutics | AV-1980R | AD | Exhibits immunostimulant properties to produce antibodies against tau protein. DNA vaccine developed based on MultiTEP platform. |
|  |  | AV-1991CP | AD | Chemically modified protein vaccine, targeting p-tau protein at serine (396-404). |
|  |  | AV-1992CP | AD | Chemically modified protein vaccine candidate. It acts by stimulating the immune response against acetylated tau protein (Tau k174). |
|  | Nuravax Inc. | AV-1959R + AV-1980R (Duvax) | AD | Acts by targeting both Aꞵ and NFTs and is being developed based on MultiTEP carrier platform. |
|  | Vaxxinity | Anti-tau | AD, CTE | Induces immune response against mutated tau. |
| Synthetic peptide | Priavoid GmbH | PRI-200 | AD, tauopathies | A MAPT inhibitor. |
|  | Weizmann Inst. Of Science | Peptides | AD | Mixture of two peptide that inhibit APP and MAPT to reduce plaque formation, and soluble protein levels in the brain to improve cognitive function. |
| Unknown | Medifron DBT | Drug to inhibit tau for AD | AD | Tau phosphorylation inhibitor. |
|  | Ovensa | Drugs to inhibit Aꞵ protein and tau for AD | AD | Act as a tau protein and amyloid beta-protein inhibitor. |
| Vaccine | Prothena Corp. | PRX-123 (Aꞵ-tau vaccine) | AD | Generates polyclonal responses against essential epitopes with the N-terminal of Aꞵ and specific regions of tau to promote amyloid clearance and blockade of tau transmission. |
|  | Univ. of Texas Medical Branch at Galveston | Vaccine to target tau for AD and PD | AD, PD | Induces immune response against mutated tau. |

**Supplementary Table 3:** Tau therapeutics in discovery (as of February 28, 2023).

| **Molecule Type** | **Company Name** | **Drug Name** | **Indication** | **Mechanism of Action** |
| --- | --- | --- | --- | --- |
| Antibody | Neural Stem Cell Institute | Antibodies to inhibit tau | CBD, cortical basal ganglionic degeneration, PSP | Bi-functional intrabodies engineered to target tau. |
|  | Oligomerix | Antibody to inhibit tau oligomer | AD | Tau oligomer specific antibody fragments that act by targeting the tau oligomer aggregate. |
| Aptamer | Chimerna Therapeutics | Aptamer to inhibit tau | AD | Circular RNA aptamer (RNA circles resist degradation) that acts by targeting MAPT. |
| Conjugate vaccine | Tria Bioscience | Vaccine to target tau | AD | Induces antibody immune responses against tau protein altered tau protein resulting in its neutralization. |
|  |  | Vaccine to target tau | TBI | Induces antibody immune responses against tau protein altered tau protein resulting in its neutralization. |
| Fusion protein | Imnewrun Bioscience | INR-303 | NDs | Antibody conjugated with blood brain barrier penetrating peptide and delivered using Transmab technology. It acts by inhibiting tau protein. |
|  | Proclara Biosciences | NPT-189 | AD | Targets Aβ, tau, transthyretin and ⍺-syn proteins. |
|  |  | Fusion proteins to inhibit ABPP, MAPT and NACP for amyloidosis and NDs | NDs | Aβ peptide, tau proteins and ⍺-syn protein inhibitor. |
| Gene therapy | Neurimmune Holding AG | AAV hmAb | AD, tauopathies | AAV vectors to express human antibodies against MAPT. |
| mAb | ADEL | ADELY-02 | Dementia associated with AD | Acts by targeting tau. |
|  | Aeton Therapeutics | AT-001 | AD | Humanized anti-acetylated tau antibody, targeting acetylated tau. |
|  | Covalent Bioscience | mAbs to inhibit MAPT | AD | Catalytic monoclonal antibodies (Catabody), act by targeting misfolded tau. |
|  | Gen2 Neuroscience | Therapeutics (antibodies) | Dementia | Specifically, targets the toxic forms of MAPT. |
|  | Innosense | mAb conjugate to inhibit tau for AD (Cyphonatol) | AD | Tau oligomer antibodies conjugated on photonic nanoparticles (pNP) which are loaded with indocyanine green (ICG) and curcumin (Cur). It acts by targeting tau aggregates. |
| Small molecule | Anhorn Medicines | Small molecule to inhibit tau | FTD, PSP, AD | Protein degradation. With BIGPRO® molecules, the target protein can be brought to close proximity of E3 complex for ubiquitination and is subsequently destroyed by the proteasome. |
|  | Anima Boitech | Small molecules to inhibit MAPT | AD | Undisclosed, but based on the platform technology of the company (small molecule drugs that selectively control mRNA translation) it is likely they are tau translation inhibitors. |
|  | Aquinnah Pharmaceuticals | Small molecules for the treatment of AD | AD | The compounds eliminate stress granules. |
|  | Korea Inst. Of Science and Technology | HN21C0315 | PSP, tauopathies | Acts by inhibiting 4R-tau oligomerization, and also promotes the secretion of neurotransmitters and stimulation of cholinergic transmission. |
|  | Oligomerix | OLX-23010 | NDs | Small molecule that acts as MAPT inhibitor. |
|  | Prazer Therapeutics | PRX-302 | AD | A protein degrader of MAPT through the ubiquitin-proteasome system. |
|  | PTC Therapeutics | MAP-Tau | Tauopathies | Splicing of pre-mRNA of MAPT leading to nonsense mediated decay of mRNA, thereby lowering tau. |
|  | Takeda Pharmaceutical | Small molecules to inhibit MAPT | AD | Undisclosed |
|  | TauRx Therapeutics | Small molecule 1 to inhibit tau | AD | Acts by dissolving misfolded tau proteins and reducing neurofibrillary tangles. |
|  | Univ. of Dundee | Small molecules to inhibit tau for AD | AD | Act by dissolving misfolded tau proteins and reducing neurofibrillary tangles. |
|  | Ventus Therapeutics | VENT-03 (cGAS) | NDs | Acts as a cGAS inhibitor. cGAS activation leads to a type I interferon response, which is implicated in a broad range of diseases, including neuroinflammatory and neurodegenerative diseases. |
|  | Wren Therapeutics | Tau | Tauopathies | Acts as a MAPT inhibitor. |
| Unknown | LSL Neuroscience | Memrin | AD | Acts as a tau inhibitor. |
|  | PharmaKure | PK-061 | AD | Undisclosed |

**Supplementary Table 4:** Inactive tau therapeutic programs (as of February 28, 2023)

| **Molecule Type** | **Company Name** | **Drug Name** | **Indication** | **Mechanism of Action** |
| --- | --- | --- | --- | --- |
| Antibody | NsGene A/S (Inactive) | Antibody to inhibit tau for CBD | CBD | Tau antibody (no further details available). |
|  | Prothena | Antibodies to inhibit tau for AD and tauopathies | AD, tauopathies | Reported to block the binding of tau to cells, cell-to-cell transmission, and downstream functional effects. |
| Antisense RNAi oligonucleotide | Univ. of Iowa | Antisense RNAi oligonucleotides to inhibit amyloid precursor protein and tau for AD | AD | Causes the down regulation of mutant Tau (V337M) and mutant amyloid precursor protein (Swedish double mutation, K670N/M671L), thereby exhibiting therapeutic intervention. |
| Biologic | Proclara Biosciences | NPT-002 | AD, PD | Disrupts aggregation of abnormally folded amyloid-beta and tau proteins. |
|  |  | NPT-001 | AD, PD | It is a bacteriophage. Disrupts and clears multiple misfolded proteins such as beta amyloid (amyloid precursor protein) and tau, as well as ⍺-syn. |
| Fusion protein | Proclara Biosciences | NPT-289 | AD, PD | Fusion protein of human immunoglobulin and a natural bacteriophage derived protein fragment. It acts by targeting Aꞵ (amyloid precursor protein), ⍺-syn, prion and tau protein. |
| Gene therapy | Voyager Therapeutics | VYTAU-01 | AD, dementia | AAV gene therapy vectorized version of an anti-tau mAb. |
| mAb | Biogen | BIIB-076 (NI-105) | AD | mAb that acts by targeting extracellular tau proteins. The anti tau program is based on reverse translational medicine technology. |
|  | Bristol-Myers Squibb | mAbs to inhibit tau for NDs | AD, dementia, PSP, tauopathies | It is an mAb developed based on induced pluripotent stem cell (iPSC) technology. |
|  |  | IPN-002 | AD | The drug candidate is a mAb, a parental version of the lead candidate IPN-007. IPN-002 selectively targets and binds to eTau outside of the cell. |
|  | Innovative NeuroTechnologies | INT-1 | Tauopathies | Adenovirus delivering mAb (INT-1). INT-1 is a phosphatase activating domain (PAD) derived mAb which acts by targeting N-terminal of tau protein. |
|  |  | INT-2 | Tauopathies | Adenovirus delivering mAb (INT-2). The drug candidate is a phosphatase activating domain derived mAb which acts by targeting N-terminal of tau protein. |
|  |  | INT-3 | Tauopathies | Delivers mAbINT-3 which binds to tau protein phosphorylated on tyrosine-18. INT-3 binds to tau and inhibits its hyperphosphorylation thus inhibiting its accumulation. |
|  | Intellect Neurosciences | T-01OX2 | AD, dementia | A conjugated mAb that targets a neoepitope that is formed following the cleavage of intact tau protein by enzymes known as "executioner" caspases to yield the smaller delta tau, which is toxic and prone to form tangles inside nerve cells and occurs early in the pathogenesis of the disease. |
|  | LA Cell | mAb to inhibit tau for CNS disorders | AD, dementia, PD, TBI | Intracellular tau mAb. |
| Recombinant protein | Proclara Biosciences | NPT-005 | Amyloidosis | Clears pathologic amyloid protein aggregates. It acts as amyloid-beta peptide, tau proteins and ⍺-syn protein inhibitor. |
| Small molecule | ALS Biopharma | Small molecule to inhibit tau for AD and tauopathies (11S-Myricanol; (+)-S-myricanol) | AD, tauopathies | Promotes tau degradation through an autophagic mechanism. |
|  | Bristol-Myers Squibb | BMS-241027 (KOS-862, Epothilone D) | AD | It is a natural polyketide compound isolated from the myxobacterium Sorangium cellulosum (also known as desoxyepothilone B). Epothilone D binds to tubulin and inhibits the disassembly of microtubules, resulting in the inhibition of mitosis, cellular profileration and cell motility. |
|  | Cantex Pharmaceuticals | Azeliragon (PF 04494700; RAGE antagonist; TTP 488) | AD | Inhibits the receptor for advanced glycation end products (RAGE). RAGE antagonists act by influencing the transport of beta-amyloid into the brain, by manipulating inflammatory mechanisms, or prevents tau hyperphosphorylation. |
|  | Centre National de la Recherche Scientifique | Small molecule to inhibit tau for AD | AD | Undisclosed mechanism of inhibition of tau. |
|  | Fulcrum Therapeutics | Small molecule to inhibit MAPT for AD | AD | Inhibitor of tau. |
|  | GliaCure | GC021109 | AD | Acts as an agonist of P2Y6 receptors and stimulates microglial phagocytosis. It also reduces the levels of IL-13 and IL-4, IL-12, and IL-17, which are key pro-inflammatory molecules. |
|  | Merck & Co. | MK-8719 | PSP | An O-GlcNAcase (OGA) inhibitor. The inhibition of OGA reduces the levels of abnormal or hyperphosphorylated tau in the brain thereby protecting brain cells and preventing the formation of the neurofibrillary protein tangles and reduces neurodegeneration. |
|  | Neuro-Hitech (Inactive) | NHT-0012 | AD | Second-generation compound that blocks the aggregation of beta-amyloid (amyloid precursor protein) and tau-proteins. |
|  | Neuronautics (Inactive) | NNI-5 | AD | Inhibits tau filament formation. |
|  |  | NNI-3 | AD | Inhibits tau filament formation. |
|  | Neuvivo Inc. | NP001 (sodium chlorite) | AD | An approach that regulates macrophage activation and transforms macrophages from a neurotoxic state to a neuroprotective state, normalizing the cellular environment of nerve cells. It targets macrophages in the blood and microglia, causing them to switch from an active "attack" mode to a "protective" mode. |
|  | ProteoTech, (Inactive) | PTI-51CH3 backup | PSP | Causes the disruption of tau protein tangles. |
|  |  | PTI-51CH3 | AD, PSP | Causes the disruption of tau protein tangles. |
|  |  | PTI-80 backup | AD | Aꞵ A4 and MAPT inhibitor. |
|  |  | PTI-80 | AD, PSP | Inhibits and reduces tau protein from forming PHFs. |
|  | reMYND NV | ReS-8T | AD | Tau protein inhibitor. |
|  |  | ReS-10T | AD | Tau protein inhibitor. |
|  | Ryvu Therapeutics SA | SEL-141 | AD, tauopathies | Dual specificity towards tyrosine phosphorylation regulated kinase 1A and MAPT inhibitor. |
|  | Samus Therapeutics | Icapamespib (PU-AD; PU HZ151) | AD | Heat shock protein 90 inhibitor to prevent aggregation and tau hyperphosphorylation. |
|  | TauRx Therapeutics | TRx-0014 (Rember) | AD | Targets the tau aggregation pathway possibly preventing NFTs. |
|  | Treventis Corp | TRV-101 | AD | RV-101 elicits its activity by inhibiting Aꞵ peptide and MAPT. |
|  |  | TRV-217 | AD | Inhibits APP and tau aggregation. |
|  |  | TRV-1140 | AD | Inhibits Aꞵ peptides and tau aggregation. |
|  |  | TRV-1387 | AD | Inhibits Aꞵ-40, Aꞵ-42 and tau-441 aggregation and prevents tau NFT formation. |
|  | Univ. of Michigan | Small molecules to inhibit tau for AD and tauopathies | AD, tauopathies | The compounds hit a tau target by correcting tau loss of function, thereby stabilizing microtubules, and offsetting the loss of tau due to its formation into NFTs. |
| Subunit vaccine | Axon Neuroscience | AADvac-1 | AD | AADvac-1 stimulates immune systems to produce antibodies that clear amyloids and prevent accumulation and deposition of NFTs. It also may stimulate the immune system to produce antibodies against tau. |
| Synthetic peptide | Coronis NeuroSciences | Davunetide (AL 108; AL 208; CP 201) | AD, cognitive impairment associated with schizophrenia, dementia, MCI, PD, schizophrenia | Interacts with microtubule end binding proteins (SxIP motif), stabilizes, and repairs microtubules and restores neuronal structure and function. |
|  | Declion Pharmaceuticals | DPC-016 | PSP | DP-C016 formulated with PLA i-particles induces antibodies against p-tau without the inflammation responsible for harmful encephalopathy and microhemorrhage. |
| Unknown | Annovis Bio Inc. | ANVS-405 | AD | Inhibits the production of neurotoxic porteins that are derived from APP, α-synuclein and tau. |
|  | Proclara Biosciences | Drug to inhibit tau for tauopathy | Tauopathies | Acts by inhibiting the accumulation of tau. |
| Vaccine | Intellect Neurosciences | RV-02 | AD | The drug candidate stimulates the immune system to produce antibodies against tau. |
|  |  | RV-03 | AD | Peptide vaccine which induces an immune response against both beta amyloid and delta tau. |

**Supplementary Table 5:** Recently discontinued tau therapeutic programs (as of February 28, 2023)

| **Company Name** | **Drug Name** | **Indication** | **Latest Stage Reported** | **Molecule Type/ Mechanism** |
| --- | --- | --- | --- | --- |
| AbbVie Inc. | Tilavonemab (ABBV-8E12; C2N-8E12) | AD, PSP | Ph. II in AD and PSP | mAb, recombinant IgG4 antibody that targets tau proteins. |
| Biogen Inc. | Gosuranemab (BIIB-092) | AD, primary tauopathies: CBS, nfvPPA, sMAPT, and TES (TauBasket) (Boxer et al., 2019) | Ph. II in AD and PSP | mAb, humanized mAb that targets N-terminal tau. |
| Eli Lilly and Co. | Zagotenemab (LY 3303560) | AD | Ph. II in AD | mAb, targeting aggregated tau. |

Boxer, A. L., Qureshi, I., Ahlijanian, M., Grundman, M., Golbe, L. I., Litvan, I., Honig, L. S., Tuite, P., McFarland, N. R., O’Suilleabhain, P., Xie, T., Tirucherai, G. S., Bechtold, C., Bordelon, Y., Geldmacher, D. S., Grossman, M., Isaacson, S., Zesiewicz, T., Olsson, T., … Dam, T. (2019). Safety of the tau-directed monoclonal antibody BIIB092 in progressive supranuclear palsy: a randomised, placebo-controlled, multiple ascending dose phase 1b trial. The Lancet Neurology, 18(6), 549–558. https://doi.org/10.1016/S1474-4422(19)30139-5

**Supplementary Figure 1:** Therapeutic approaches in development that indirectly target tau.


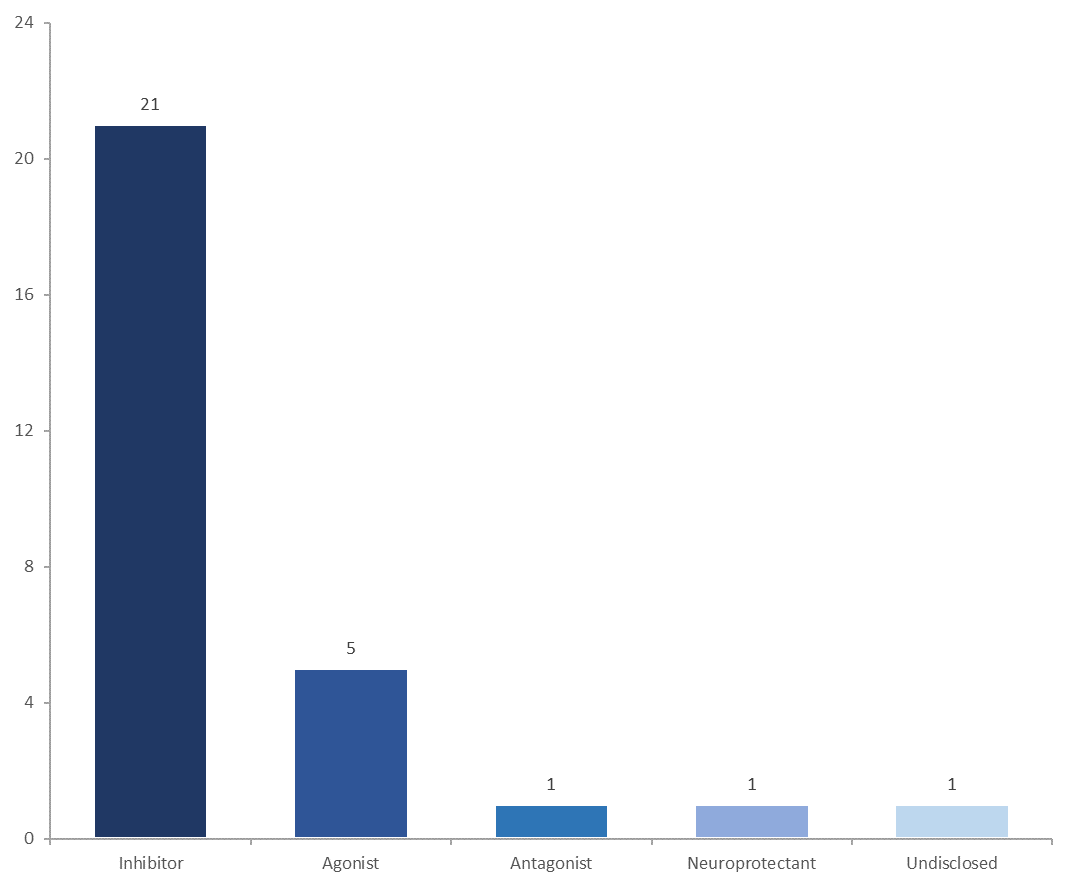


**Supplementary Figure 2:** Mechanism of action of drugs in clinical trials.


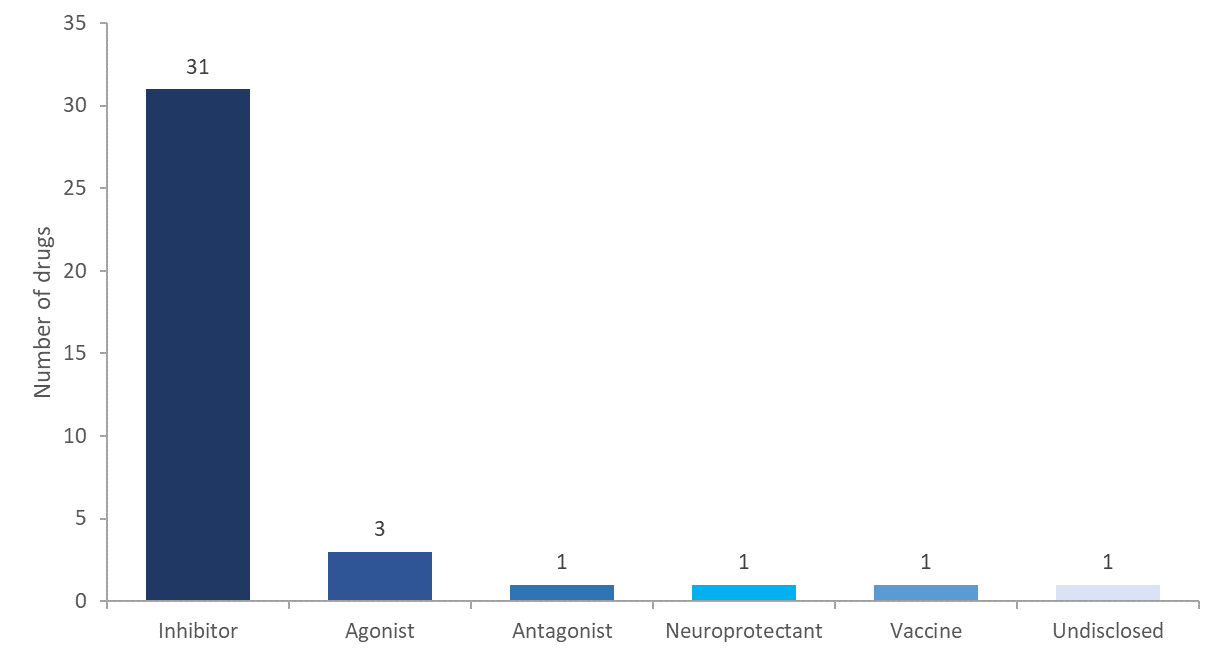


**Supplementary Figure 3:** Routes of administration for tau therapeutics in clinical trial phase I, II, and III.


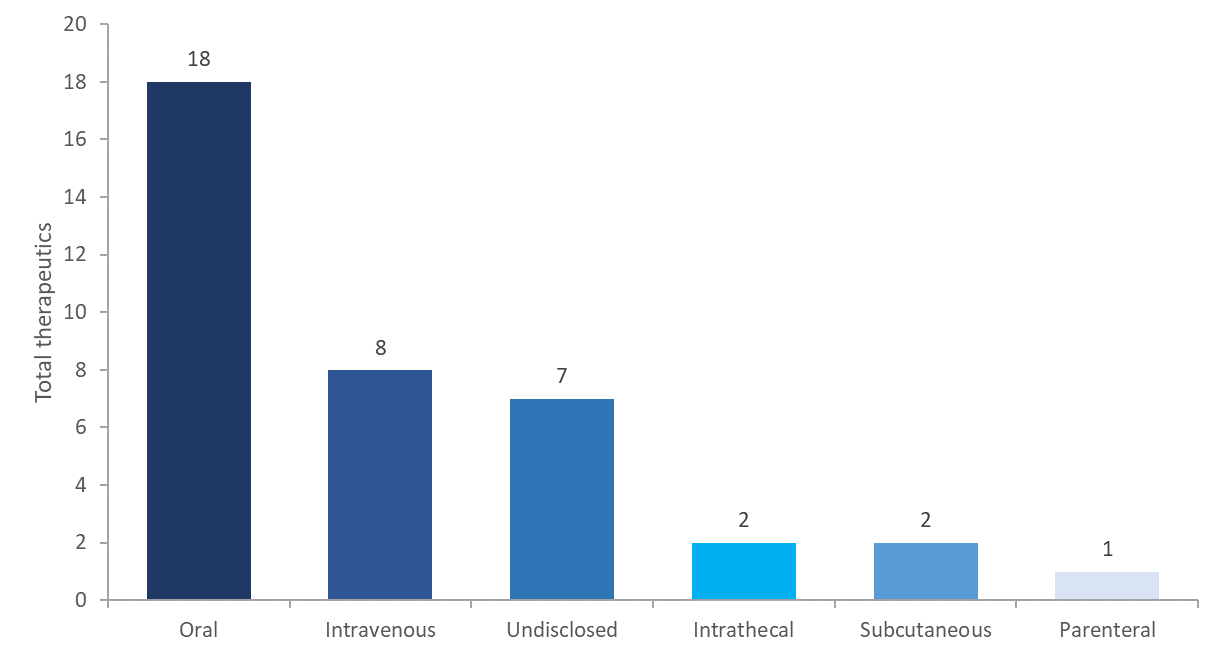

Supplement: Supplementary file 1 — Additional file 1: Supplementary Table 1. Classification of therapeutic targets for tauopathies. Supplementary Table 2. Tau therapeutics in preclinical development (as of February 28, 2023). Supplementary Table 3. Tau therapeutics in discovery (as of February 28, 2023). Supplementary Table 4. Inactive tau therapeutic programs (as of February 28, 2023). Supplementary Table 5. Recently discontinued tau therapeutic programs (as of February 28, 2023). Supplementary Figure 1. Therapeutic approaches in development that indirectly target tau. Supplementary Figure 2. Mechanism of action of drugs in clinical trials. Supplementary Figure 3. Routes of administration for tau therapeutics in clinical trial phase I, II, and III. [file 13195_2023_1321_MOESM1_ESM.docx]
